# Hypoxia-induced mitochondrial stress granules

**DOI:** 10.1038/s41419-023-05988-6

**Published:** 2023-07-19

**Authors:** Chun-Ling Sun, Marc Van Gilst, C. Michael Crowder

**Affiliations:** 1 Department of Anesthesiology and Pain Medicine, University of Washington School of Medicine, Seattle, Washington 98109 USA; 2 Mitochondrial and Metabolism Center, University of Washington School of Medicine, Seattle, Washington 98109 USA; 3 Department of Genome Science, University of Washington School of Medicine, Seattle, Washington 98109 USA

**Keywords:** Mitochondria, Cell death, Stroke

## Abstract

Perturbations of mitochondrial proteostasis have been associated with aging, neurodegenerative diseases, and recently with hypoxic injury. While examining hypoxia-induced mitochondrial protein aggregation in *C. elegans*, we found that sublethal hypoxia, sodium azide, or heat shock-induced abundant ethidium bromide staining mitochondrial granules that preceded evidence of protein aggregation. Genetic manipulations that reduce cellular and organismal hypoxic death block the formation of these mitochondrial stress granules (mitoSG). Knockdown of mitochondrial nucleoid proteins also blocked the formation of mitoSG by a mechanism distinct from the mitochondrial unfolded protein response. Lack of the major mitochondrial matrix protease LONP-1 resulted in the constitutive formation of mitoSG without external stress. Ethidium bromide-staining RNA-containing mitochondrial granules were also observed in rat cardiomyocytes treated with sodium azide, a hypoxia mimetic. Mitochondrial stress granules are an early mitochondrial pathology controlled by LONP and the nucleoid, preceding hypoxia-induced protein aggregation.

## Introduction

The effects of low oxygen levels (hypoxia) on mitochondrial function and homeostasis have been intensely studied with the potential that early mitigation of mitochondrial pathology may prevent the cascade of events leading to cell death in disease processes such as stroke and myocardial infarction. Unfortunately, pharmacological efforts at reducing mitochondrial dysfunction or its consequences have failed to produce clinically meaningful improvements in patient outcomes [1, 2]. Although less well-studied, other mitochondrial pathological processes not targeted thus far in clinical trials may nevertheless be critical to the hypoxic cell death cascade. Mitochondrial fission is induced by hypoxia; however, the role of this excess fission in cell death is controversial, and genetic or pharmacological manipulation of fission or fusion has not yielded a consistent effect on ischemic or hypoxic cell death [3–5]. Mitochondrial proteostasis is also disrupted by hypoxia but the mechanisms underlying these effects are only beginning to be elucidated [4, 6–11]. Mitochondrial protein synthesis, import, folding, degradation, and removal are all energy-consuming processes that are impaired by hypoxia. The relative contribution of disruption of these processes to mitochondrial failure and cell death is unknown.

In *C. elegans*, an early pathological feature of hypoxia is an increase in intramitochondrial protein misfolding [9–11]. Sublethal hypoxia, from which *C. elegans* fully recovers, induces a homeostatic response to mitochondrial misfolded proteins, called the mitochondrial unfolded protein response UPR^mt^. Activation of the UPR^mt^ in *C. elegans* results in increased expression of mitochondrial chaperones, proteases, import machinery components, ROS detoxifying enzymes, and multiple other proteins that can be adaptive during and after a hypoxic insult [12]. Activation of the *C. elegans* UPR^mt^ is often accompanied by lifespan extension, and UPR^mt^ activation in both *C. elegans* and in mammals has been found to be cytoprotective [6, 7, 9]. However, in *C. elegans*, induction of the UPR^mt^ is neither necessary nor sufficient for protection from severe hypoxic cellular injury nor for lifespan extension [7].

Consistent with inducing protein misfolding, sublethal hypoxia in *C. elegans* produces protein aggregates visible within the mitochondria as electron-dense granules, as fluorescently-tagged protein aggregates, and as the mitochondrial dye TMRE-staining granules [7, 8]. The nature of these hypoxia-induced mitochondrial protein aggregates (HIMPA) is poorly defined. Several fluorescently tagged proteins produce HIMPA. However, the number of proteins that form an aggregate, what controls aggregate formation, whether aggregates form in an orderly or stochastic process, and whether other macromolecules might be contained within the aggregates are all questions remaining to be answered. Whether HIMPA occurs in other cells besides those in *C. elegans* is also unknown. Here we investigate whether HIMPA contains nucleic acid.

## Results

### Hypoxia induces EtBr-staining granules in mitochondria

**Fig. 1:**
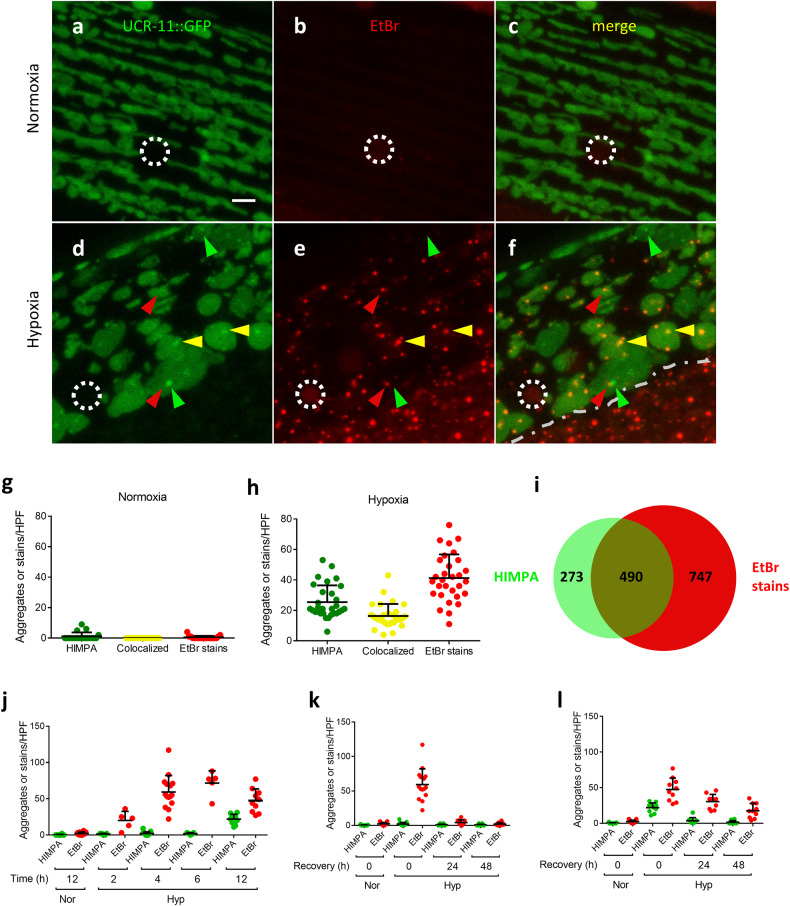
Hypoxia induces EtBr staining granules in mitochondria. **a**–**f** Examples of UCR-11::GFP expressed in muscle and EtBr staining immediately after normoxia (**a**–**c**) or 12 h hypoxia (**d**−**f**). UCR-11::GFP muscle HIMPA (green arrowhead), EtBr stain (red arrowhead) and co-localization of both (yellow arrowhead), nucleolus (white dashed circle). The gray dashed line in **f** demarcates the edge of the muscle; the EtBr granules outside the muscle are not colocalized with the muscle-specific UCR-11::GFP expression. Scale bar = 3 μm. **g, h** Quantification of HIMPA, EtBr granules and colocalization after normoxia **(g)** and after hypoxia **(h)**. Mean ± SD were obtained from 19 (normoxia) or 30 (hypoxia, 12-13 h) individual animals from 5 independent experiments. **i** Level of colocalization of UCR-11::GFP HIMPA and EtBr stains. Data were obtained from 30 individual animals from 5 independent experiments. **j** *gcIs46*[UCR-11::GFP] animals pre-stained with EtBr and scored immediately after indicated hours of normoxia (Nor) or hypoxia (Hyp). UCR-11::GFP HIMPA (green dots) and EtBr staining granules (red dots) scored. Mean ± SD were obtained from 5 (2 and 6 h hypoxia) or 10-15 (4 and 12 h hypoxia) individual animals from 1-3 independent experiments. **k, l** Recovery kinetics of UCR-11::GFP HIMPA and EtBr staining granules after 4 h **(k)** or 12-15 h hypoxia **(l)**. Mean ± SD were obtained from 10 (24 h or 48 h recovery) or 15 (0 h recovery for normoxia or hypoxia) individual animals from 2-3 independent experiments.

Utilizing an animal expressing a UCR-11::GFP mitochondrial fusion protein in body wall muscle, mitochondria were visualized after normoxic and hypoxic incubations. As previously reported [8, 11], UCR-11::GFP was uniformly expressed throughout the mitochondria, and no aggregates were apparent after normoxic incubation (12 hours of 20.9% oxygen@26.5 °C) (Fig. 1a, c, g). After a sublethal hypoxic incubation (12 hours of <0.3% oxygen@26.5 °C), UCR-11::GFP aggregates (HIMPA) were visible within a substantial fraction of mitochondria (Fig. 1d, f, h). Curiously, HIMPA are almost exclusively visible as single aggregates within each mitochondrion. There are two characterized mitochondrial structures that tend to be single and localized like HIMPA: 1) the nucleoid, containing mitochondrial DNA, and 2) mitochondrial RNA granules [13, 14]. To test whether HIMPA includes nucleic acids, we transferred live worms onto agar plates containing ethidium bromide (EtBr), a nucleic acid dye that stains both DNA and RNA. EtBr faintly stained the nucleolus of normoxic worms but there was no obvious cytoplasmic or mitochondrial granules (Fig. 1b, c, g). Hypoxia-induced many EtBr-staining granules in mitochondria (Fig. 1e, f, h and Supplementary Fig. 1). About 40% of the EtBr-staining granules colocalized with the UCR-11::GFP aggregates, and about 64% of the UCR-11::GFP aggregates colocalized with the EtBr-staining granules (Fig. 1h, i). Thus, at least at the level of detection of confocal microscopy, not all HIMPA contain EtBr-staining granules; likewise, not all EtBr-staining granules contain HIMPA but there is significant colocalization.

### EtBr-staining granules occur earlier, persist longer than HIMPA and caused by other stressors

To determine the temporal relationship between HIMPA and the EtBr-staining granules, we assessed the duration of hypoxic exposure required for their appearance and how long after reoxygenation they persisted. The EtBr granules were visible after as little as two hours of hypoxia (Fig. 1j). For context, *C. elegans* are still moving after two hours under these conditions and have no apparent sequelae after recovery [15]. As previously reported [8], HIMPA required much longer hypoxic incubation and were not detectable until after 12 hours of hypoxia (Fig. 1j); after twelve hours of hypoxia, a significant fraction of animals die and most of the remainder have permanent behavioral deficits, arguing that HIMPA is likely a premorbid hypoxic pathology [15]. The kinetics of granule disappearance depended on the severity of the hypoxic insult. After a 4-hour hypoxic incubation, the EtBr-staining granules disappeared after a 24-hour recovery (Fig. 1k). After 12-15 hours of hypoxia, the EtBr-granules persisted much longer and were still detectable, albeit at a lower level, even 48 hours after recovery from hypoxia (Fig. 1l). HIMPA levels were near baseline after a 24 h recovery (Fig. 1l). These results demonstrated that EtBr-staining granules occur earlier and persist longer than HIMPA, indicating that HIMPA does not drive the formation or persistence of the EtBr-staining granules.

**Fig. 2:**
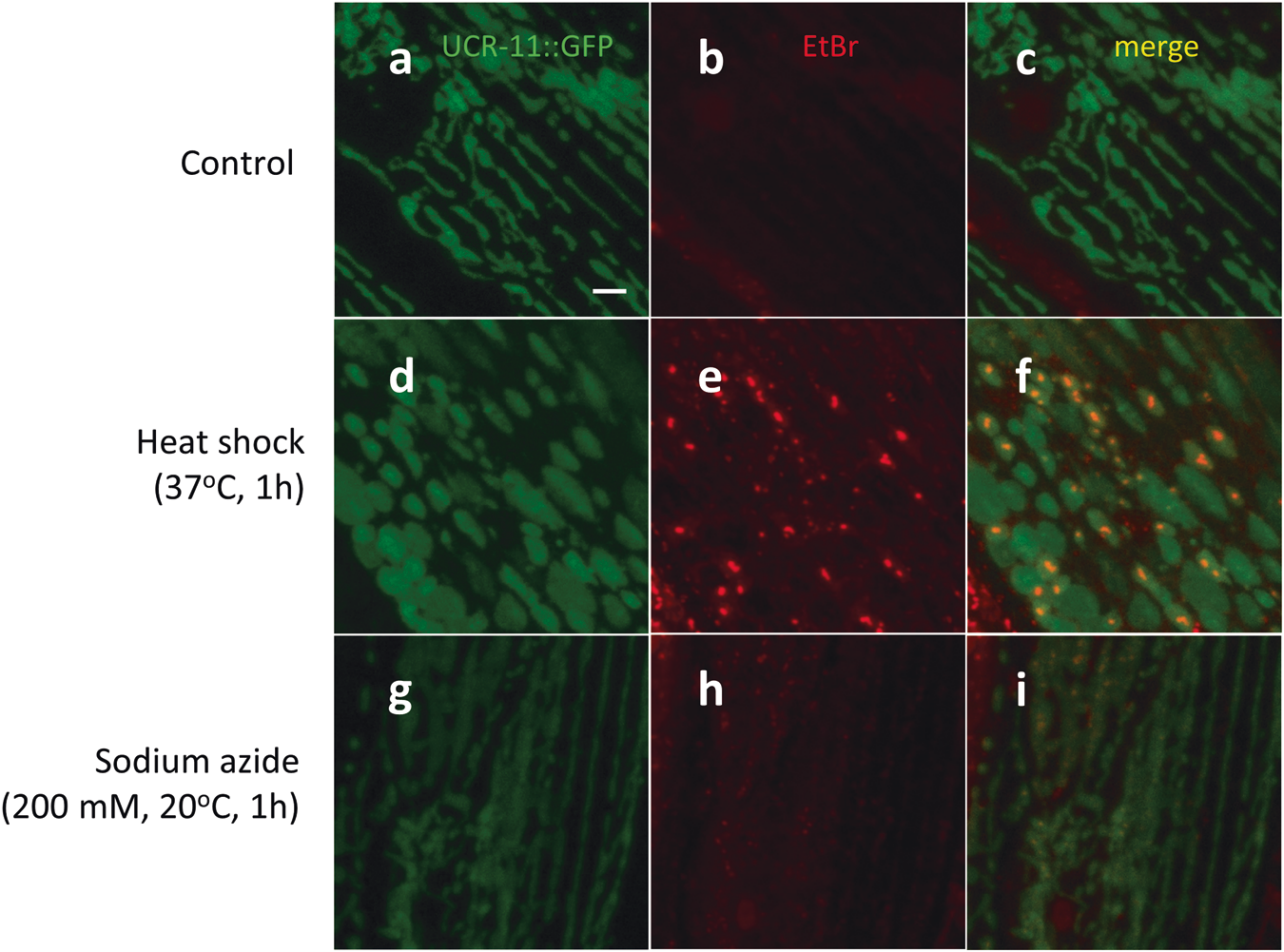
EtBr staining granules are induced by other forms of cellular stress. **a**–**i** Examples of UCR-11::GFP (green) and EtBr staining granules (red) in *gcIs46* animals after 1 h incubation at 20 ^o^C (Control, **a**–**c**), at 37 ^o^C (heat shock, **d**–**f**) or with sodium azide at 20 ^o^C (200 mM, **g**–**i**). Scale bar = 3 μm.

We investigated the effect of other forms of cellular stresses on EtBr-staining granules. Heat shock (37 °C, 1 h) produced EtBr-staining granules, but not HIMPA (Fig. 2a–f). Sodium azide, a chemical hypoxia mimetic, also led to EtBr-staining granules, but not HIMPA (Fig. 2g–i). These data demonstrate that in addition to hypoxia, other mitochondrial stresses (heat and sodium azide) can induce EtBr-staining granules. The data further demonstrate that EtBr-staining granules and HIMPA are distinct mitochondrial pathologies. Given that multiple forms of cellular stress induce EtBr-staining granules, we will refer to them as mitochondrial Stress Granules (mitoSGs).

To determine whether expression of the aggregation-prone UCR-11::GFP fusion protein in the mitochondria was artificially inducing the mitoSGs, we visualized body wall muscle mitochondria with a distinct non-aggregating reporter *ccIs4251* (*myo-3p:: mitoGFP*) [16] and observed a similar level of mitoSGs in *ccIs4251* and in UCR-11::GFP animals (Supplementary Fig. 2a-f). MitoSGs were not specific to muscle mitochondria and were observed in both intestinal cells and touch receptor neurons (Supplementary Fig. 2g–r).

### Molecular nature of the EtBr-staining granules

**Fig. 3:**
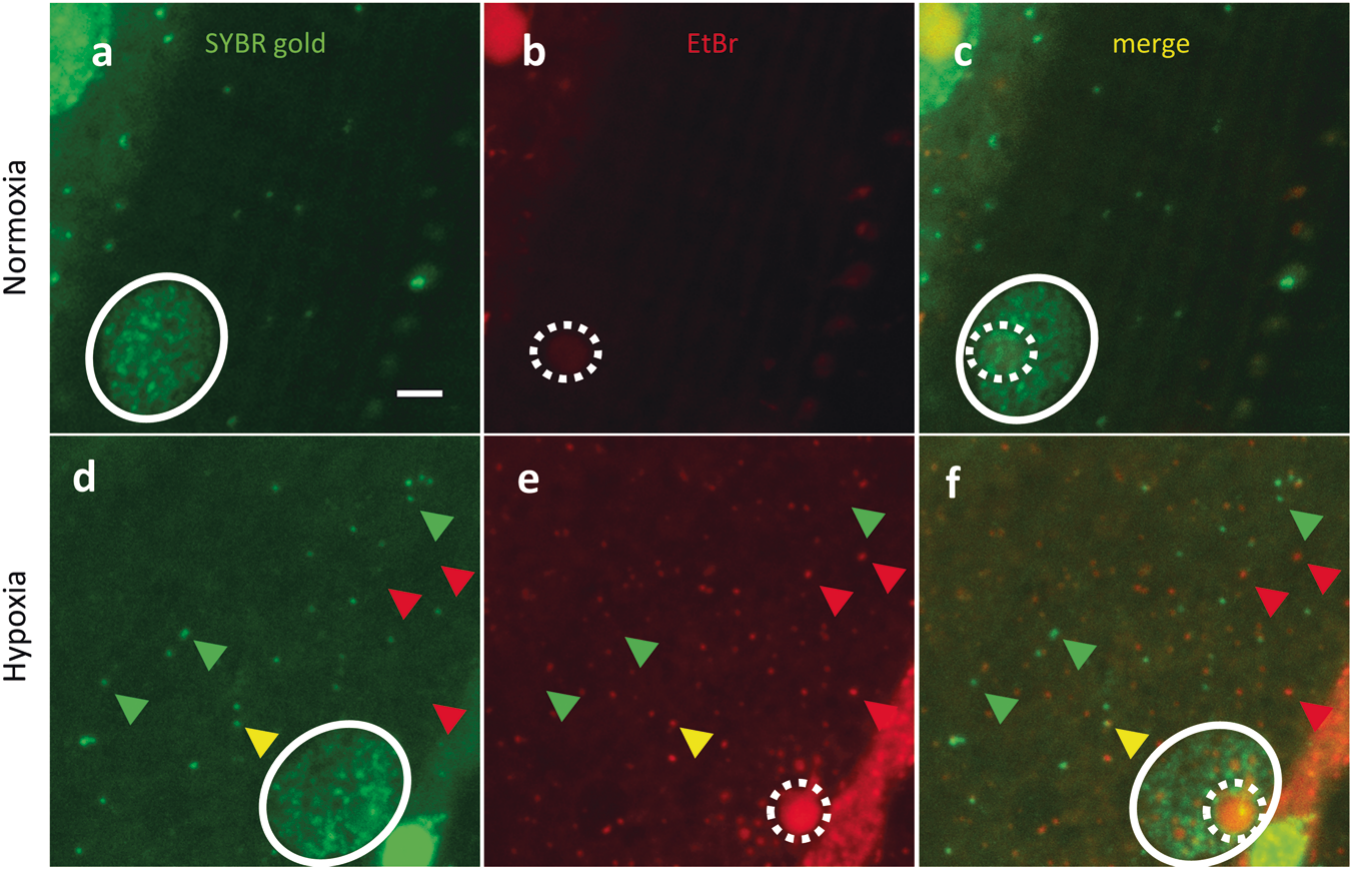
EtBr staining granules do not overlap with SYBR gold staining. **a**–**f** N2 animals were pre-stained with SYBR gold and EtBr for 24 hours and immediately imaged after 14 h normoxia **(a**–**c)** or 14 h hypoxia **(d**–**f)**. SYBR gold-stained nuclear DNA (white circle) and nucleoid DNA (green arrowhead), EtBr stained nucleolus (dotted white circle) and mitoSG (red arrowhead), co-localization of SYBR stains and mitoSG (yellow arrowhead) shown.

EtBr has been shown to label both mitochondrial DNA (nucleoid) and its RNA transcripts in human cancer cells [17–21]. To investigate if the mitoSGs were composed of DNA, RNA, or both, we applied SYBR gold, a DNA-preferring dye [22], in a co-staining assay with EtBr in worm body wall muscle. In normoxia, SYBR gold stained nuclear DNA and extra-nuclear puncta that are presumed to be mitochondrial nucleoid DNA in body wall muscle of wild-type N2 (Fig. 3a), whereas EtBr stained an oval-shaped structure within the nucleus consistent with the nucleolus and a few extra-nuclear puncta that did not colocalize with the SYBR gold spots (Fig. 3b, c). Hypoxia did not clearly change SYBR gold staining despite the induction of mitoSGs (Fig. 3d, e) SYBR gold and EtBr displayed rare colocalization (Fig. 3f), indicating that EtBr is not primarily staining DNA and that these granules most likely contain RNA.

To test further if RNA is the main target of staining by EtBr in vivo, we determined whether DNase I or RNase A could reduce EtBr-staining. Attempts at performing the fixation and permeabilization of body wall muscle after hypoxia required for DNase and RNase treatment resulted in poor EtBr staining and loss of visualization of the mitochondrial granules. Instead, we examined ex vivo dissected gonads where fixation and permeabilization is routine for antibody staining. We utilized a strain where a histone GFP::H2B fusion protein was expressed in germ cells to visualize chromatin. The germ cell chromatin was well-visualized with GFP::H2B after both normoxic and hypoxic incubations (Supplementary Fig. 3a, d). SYBR gold stained in a chromatin pattern similar to GFP::H2B, confirming its DNA preference (Supplementary Fig 3j, m). EtBr stained the nucleolus, which was more visible after hypoxia, but staining of chromatin was not apparent after normoxic or hypoxic incubation in either GFP::H2B-labelled or SYBR gold-labelled germ cells (Supplementary Fig. 3b, e, g-i, k, n). The extranuclear EtBr staining was more diffuse than in body wall muscle and not clearly granular in gonads from animals recovering from hypoxia. SYBR gold and EtBr staining had little apparent overlap in either normoxia or hypoxia (Supplementary Fig. 3j–o). DNase I reduced SYBR gold chromatin staining in fixed and permeabilized nuclei, but had minimal, if any, effect on EtBr staining in the nucleolus (Supplementary Fig. 3p–u). RNase A reduced extranuclear staining by SYBR gold and but had no visible effect on chromatin staining; RNase A eliminated extranuclear and nucleolar EtBr staining, revealing faint chromatin staining that was not due to bleed through from SYBR-gold co-staining (Supplementary Fig. 3p–r, v–x, Supplementary Fig. 4). The lack of colocalization of EtBr staining with GFP::H2B or SYBR gold staining and elimination by RNase but not by DNase argue that RNA is the primary staining target for EtBr in vivo in *C. elegans*.

### Hypoxic protective mechanisms reduce mitoSGs

Hypoxic protective mechanisms prevent HIMPA [7]. We asked whether these same mechanisms prevent mitoSG formation. The insulin/IGF receptor pathway strongly regulates both longevity and hypoxic injury in *C. elegans* [15, 23, 24]. A hypoxic death-resistant reduction-of-function mutant of the insulin/IGF receptor gene *daf-2* was shown to block HIMPA formation [7]. We tested the role of *daf-2* in mitoSG by treating animals with *daf-2*(RNAi) prior to hypoxia and found that *daf-2*(RNAi) fully blocked mitoSG and HIMPA formation (Supplementary Fig. 5). Mutants in the translation machinery with reduced translation rate have also been shown to be hypoxia resistant [25–27]. RNAi against the arginyl-tRNA synthetase gene *rars-1* fully blocked mitoSGs as well as HIMPA (Supplementary Fig. 5). These results demonstrate that two mechanistically distinct hypoxic protective mechanisms prevent mitoSG formation, suggesting that mitoSG is an early manifestation of hypoxic cellular injury, not simply a response to hypoxia and/or temperature that is unrelated to cellular injury.

### Knockdown of nucleoid proteins blocks mitoSG formation

**Fig. 4:**
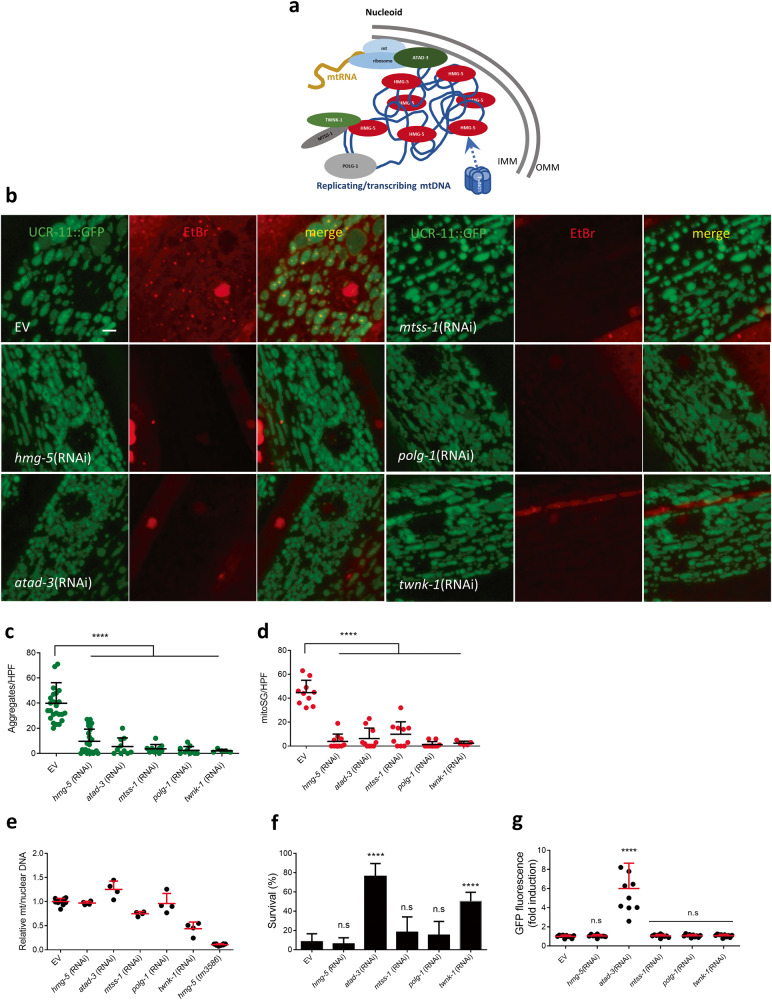
Nucleoid proteins required for mitoSG formation. **a** Schematic cartoon showing structure of nucleoid. **(b)** Examples of UCR-11::GFP HIMPA (green) and EtBr staining mitoSGs (red) scored immediately after 12 h hypoxia in animals grown in empty vector (EV), *hmg-5*(RNAi), *atad-3*(RNAi), *mtss-1*(RNAi), *polg-1*(RNAi) or *twnk-1*(RNAi). Scale bar = 3 μm. **c, d** Quantification of UCR-11::GFP HIMPA **(c)** and EtBr mitoSGs **(d)**. Mean ± SD were obtained from 5 (*twnk-1*), ~10 (other RNAis) individual animals from 1 (*twnk-1*), ~2 (other RNAis) independent experiments. **** - *p* < 0.001 by unpaired 2-sided t-test. **e** Mitochondrial DNA copy number in animals grown in empty vector (EV), *hmg-5*(RNAi), *atad-3*(RNAi), *mtss-1*(RNAi), *polg-1*(RNAi) or *twnk-1*(RNAi). *hmg-5*(*tm3586*) is a homozygote from *hmg-5*(*tm3586*)/nT1. Mean ± SD were obtained from 4 technical replicates. **f** Hypoxia survival (24 h incubation) in animals grown in empty vector (EV), *hmg-5*(RNAi), *atad-3*(RNAi), *mtss-1*(RNAi), *polg-1*(RNAi) or *twnk-1*(RNAi). Mean ± SD were obtained from 3 independent experiments with 3 technical replicates in each experiment. **** - *p* < 0.001 by unpaired 2-sided t-test, n.s. - not significant versus EV. **g** Quantification of *hsp-6*p::GFP expression in wild-type background, grown on empty vector (EV) or indicated RNAis. Mean ± SD were obtained from 10 animals. **** - *p* < 0.0001 by unpaired 2-sided t-test, n.s - not significant versus EV. Scale bar = 0.1 mm.

The nucleoid is a mitochondrial structure composed of mitochondrial DNA, ribosomal RNA, its transcripts, and associated proteins (Fig. 4a). As the sole source of mitochondrial RNA, functions of the nucleoid are likely critical to mitoSG formation. To investigate the relationship between mitoSG and nucleoid components, we tested the effect of RNAis targeting five nucleoid proteins that regulate various aspects of mitochondrial DNA replication, transcription, or ribosomal-nucleoid association [28]. Surprisingly, all five nucleoid RNAis strongly reduced both mitoSG and HIMPA (Fig. 4b–d). In *C. elegans* and other models, HMG-5/TFAM levels have been shown to positively regulate mitochondrial DNA copy number; depletion of TFAM decreases mitochondrial DNA copy number [28]. Thus, we asked if a change in mitochondrial DNA copy number might be responsible for the effect of the knockdown of *hmg-5* and the other nucleoid components on mitoSG. Although an *hmg-5* deletion mutant (*tm3586*) did strongly reduce mitochondrial DNA copy number, *hmg-5* RNAi under these conditions did not; only *twnk-1* RNAi strongly reduced mitochondrial DNA copy number (Fig. 4e). We wondered if nucleoid protein knockdown was protective against hypoxic death and therefore might indirectly block mitoSG formation. Both *atad-3* and *twnk-1* RNAis were hypoxia protective; however, *hmg-5*, *mtss-1*, and *polg-1* RNAis were not (Fig. 4f). Since activation of the mitochondrial unfolded protein response (UPR^mt^) has been shown to reduce HIMPA [7], we asked whether nucleoid protein knockdown activated the UPR^mt^. However, only *atad-3* RNAi significantly induced UPR^mt^ (Fig. 4g). Notably, high doses of EtBr have been shown to induce the UPR^mt^ [29–31]. However, the EtBr staining conditions used here (8 µg/ml EtBr for 24 h) did not induce significant UPR^mt^ activation, as measured by lack of expression of the UPR^mt^ reporter HSP-6::GFP (Supplementary Fig. 6). The mechanism underlying the requirement for these nucleoid components for mitoSGs/HIMPA formation is unclear but is not due to a change in mtDNA copy number, hypoxia resistance, or induction of the UPR^mt^.

### *lonp-1* negatively regulates mitoSG formation

**Fig. 5:**
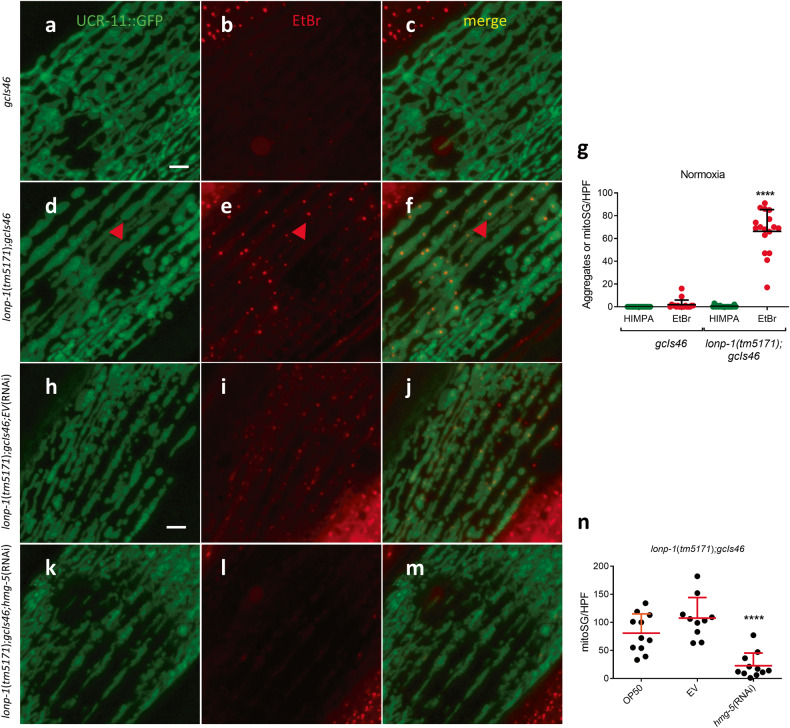
*lonp-1* loss-of-function induces HMG-5-dependent mitoSG formation. **a**–**f** Examples of UCR-11::GFP (green) and EtBr staining (red) in animals in wild-type **(a**–**c)** or *lonp-1*(*tm5171* lf) background **(d**–**f)**. **g** Quantification of UCR-11::GFP aggregates or EtBr mitoSGs under normoxic conditions in *lonp-1*(+) and *lonp-1*(lf) animals. Mean ± SD from 15 animals from 3 independent experiments. **** - *p* < 0.001 by unpaired 2-sided t-test versus *gcIs46* EtBr. **h**–**n** *lonp-1*(lf)-mediated mitoSGs are *hmg-5*-dependent. Examples of UCR-11::GFP (green) and EtBr staining granules (red) in *lonp-1*(lf)*;gcIs46* animals grown in empty vector (EV) **(h**–**j)** or *hmg-5*(RNAi) **(k**–**m)**. **n** Quantification of EtBr stained mitoSGs in animals like those in **i, l**; OP50 is a non-vector-containing control bacteria, EV=empty vector control bacteria. Mean ± SD from > 10 animals from 2 independent experiments. **** - *p* < 0.001 versus EV by unpaired 2-sided t-test. Scale bar = 3 μm.

The AAA+ mitochondrial matrix proteases LONP1 and ClpX/P are critical regulators of mitochondrial protein quality control. Both are classically defined as ATP-dependent proteases that selectively degrade misfolded proteins with accessible unstructured polypeptide segments [32, 33]. LONP1 is closely associated with the nucleoid where multiple components are its substrates [28, 32] and has both DNA and RNA binding activity [34–36]. Knockdown of LONP1 levels in yeast, Drosophila cells, and in *C. elegans* increase mitochondrial DNA content, perhaps by increasing HMG-5/TFAM levels, one of its established substrates [37–39]. Given its subcellular location and roles in mitochondrial proteostasis, DNA content, and DNA/RNA binding activity, we hypothesized that LONP-1, the *C. elegans* ortholog of LONP1, would regulate mitoSG formation. Indeed, animals with a loss of function mutation in *lonp-1* had ethidium bromide staining granules resembling mitoSGs even under normoxic conditions (Fig. 5a–g); no aggregation of UCR-11::GFP was observed. As for mitoSGs induced by hypoxia, *hmg-5*(RNAi) suppressed the *lonp-1*(lf)-mediated mitoSGs (Fig. 5h–n). *clpp-1*, encoding the *C. elegans* ortholog of ClpP, is essential for the *C. elegans* UPR^mt^ [40] and in mammals may be important for regulating mitochondrial translation and nucleoid size [41, 42]. As for *lonp-1*, a loss-of-function mutation of *clpp-1* produced mitoSG-like granules under normoxic conditions (Supplementary Fig. 7), suggesting that both proteases act in a non-redundant manner, preventing the formation of mitoSGs. Given the induction of MSG by *lonp-1*(lf) and the increase in *C. elegans* mitochondrial DNA content reported with *lonp-1*(RNAi) [39], we considered the possibility that hypoxia might also increase mitochondrial DNA content . If so, we would have to consider the possibility that MSG, at least in *C. elegans*, were the consequence of this increase in mitochondrial DNA and perhaps could contain DNA, despite the H9c2 data strongly suggesting otherwise. To test this hypothesis, we measured mitochondrial DNA content normalized to nuclear DNA content in wild-type and UCR-11::GFP animals treated with ethidium bromide or buffer and then exposed to 14 hours of hypoxia which produces abundant and long-lasting MSG. However, our data shows that hypoxia did not increase mitochondrial DNA content in any of the conditions and thus does not support the hypothesis that an increase in mitochondrial DNA content is responsible for hypoxia-induced MSG in *C. elegans* (Supplementary Fig. 8).

### mitoSGs in mammalian cells

**Fig. 6:**
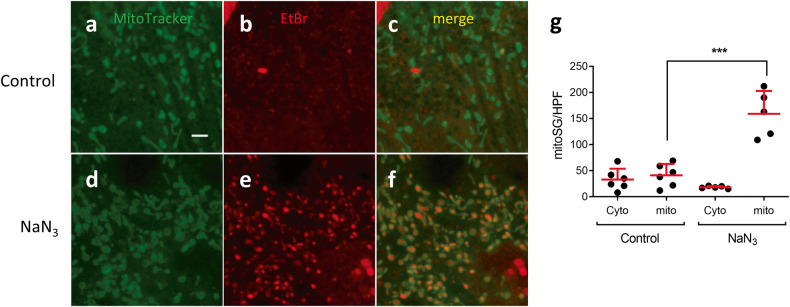
mitoSGs localize in mitochondria in mammalian cells. Control **(a**–**c)** or sodium azide (100 mM, 2 h) **(d**–**f)** treated H9c2 cells, pre-stained with MitoTracker and EtBr. **g** Quantification of mitoSG in cytoplasm (Cyto) and mitochondria (mito) as in **(a**–**f)**. Mean ± SD from 5 images. *** - *p* = 0.0003 by unpaired 2-sided t-test. Scale bar = 3 μm.

**Fig. 7:**
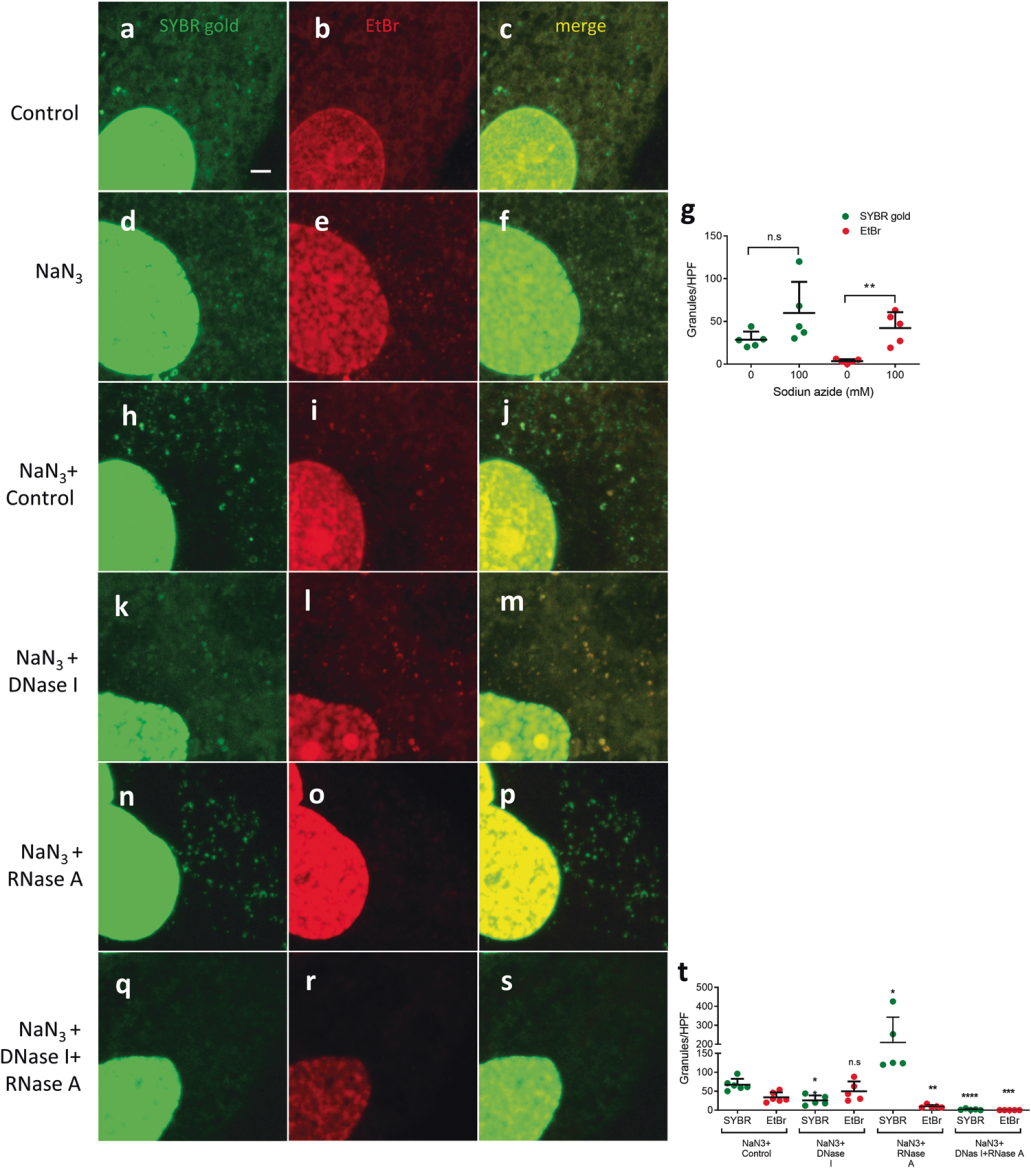
mitoSGs in mammalian cells contain RNA. Control **(a**–**c)** or sodium azide (100 mM, 3 h) **(d**–**f)** treated H9c2 cells, pre-stained with SYBR gold and EtBr. **g** Quantification of SYBR gold and EtBr staining granules as in **a**–**f**, Mean ± SD from 5 cells. ** - *p* = 0.0017 by unpaired 2-sided t-test, ns – not significant. **h**–**s** Control or sodium azide (100 mM, 3 h) treated H9c2 cells, pre-stained with SYBR gold and EtBr, were treated with NaN_3_ alone **(h**–**j**), with NaN_3_+DNaseI **(k**–**m**), with NaN_3_+RNaseA **(n**–**p)**, or with NaN_3_+DNase I+RNase A, **(q**–**s**). **t** quantification of granules as in (**h**–**s**). Mean ± SD from > 5 cells. ns-not significant, * - *p* < 0.05, ** - *p* < 0.01, *** - *p* < 0.001, **** - *p* < 0.0001 by unpaired 2-sided t-test versus NaN_3_ alone. Scale bar = 3 μm.

To investigate whether mitoSGs are present in mammalian cells, we examined a rat cardiomyocyte-derived cell line, H9c2. Using Mitotracker to label mitochondria, we treated H9c2 with sodium azide and observed widespread induction of ethidium bromide staining granules within mitochondria (Fig. 6a–g). To determine whether the EtBr staining granules were RNA or DNA, we performed SYBR gold staining and treated with DNase and RNase. Both SYBR gold and EtBr produced nuclear and extranuclear staining in control H9c2 cells (Fig. 7a–c). Sodium azide selectively increased EtBr-staining but not SYBR gold-staining granules (Fig. 7d–g). Notably, SYBR-gold granules and the EtBr granules had substantial colocalization, unlike in *C. elegans*. DNase I did not reduce EtBr granules (Fig. 7l, t). Co-treatment with DNase and RNase reduced most, if not all, SYBR gold and EtBr staining (Fig. 7q–t), whereas RNase A only eliminated the mitochondrial EtBr staining (Fig. 7n–p, t). Interestingly, RNase A treatment significantly increased the SYBR-gold granules (Fig. 7n granules (Fig. 7q–t)). These data combined with that in Fig. 6 show EtBr stains RNA-containing mitoSGs in H9c2 cells treated with sodium azide, like those observed in *C. elegans*.

## Discussion

The primary effect of severely reduced oxygen levels such as those produced by complete tissue ischemia in stroke and myocardial infarction is inhibition of the mitochondrial electron transport chain. The resultant loss of ATP production grinds to a halt a myriad of energy-dependent metabolic processes, ultimately resulting in cell death. To enhance the potential to develop effective therapies for hypoxia injury, the entire spectrum of pathological effects of hypoxia should be described and the mechanisms producing these effects elaborated. In this regard, the early effects of hypoxia before cell death processes become irreversible are particularly important. Here we describe a new pathological effect of hypoxia that occurs at an early reversible stage in the progression to hypoxia injury, persists long after reoxygenation, and is manifest as ethidium bromide staining RNA-containing granules that we have termed mitochondrial stress granules (mitoSGs).

Hypoxia has been shown to induce cytoplasmic RNA granules, so called stress granules because a variety of stresses, including hypoxia/ischemia, heat shock, and other severe cellular stresses induce them [43–47]. Cytoplasmic stress granules are composed primarily of pre-initiation complexes, including mRNAs, the 40 S ribosomal subunit, polyA-binding protein, translation initiation factors, RNA helicases, and other RNA-binding proteins [47]. Besides the core proteins, many other proteins have been found to be associated with cytoplasmic stress granules and the composition of stress granules varies by cell type and the inducing stress. While stress granules are mostly dynamic and transient, disappearing when the stress resolves, more recent evidence suggests that stress granules may persist for longer periods and may contribute to chronic more indolent conditions such as neurodegenerative disorders [47].

MitoSGs share some features with cytoplasmic stress granules. First, our data indicate that mitoSGs contain RNA. Although ethidium bromide is most commonly used as a DNA dye in vitro, our data shows that ethidium bromide strongly stains RNA in vivo in *C. elegans* with much weaker staining of DNA. Ethidium bromide staining of mitochondria has been observed in various cell lines and staining attributed to both DNA and RNA has been reported [19–21, 48]. In these studies, punctate mitochondrial staining was assumed to be DNA and diffuse staining attributed to mitochondrial RNA although this was rigorously determined for only one cell type [19]. The disappearance of mitoSGs with RNase A treatment but not DNase I treatment in H9c2 cells strongly argues that the mitoSGs contain RNA, like cytoplasmic stress granules. Second as for cytoplasmic stress granules, distinct cellular stresses, including hypoxia, heat shock, and sodium azide induce mitoSGs. Thirdly, mitoSGs often colocalize, at least at the resolution of the confocal microscope, with protein. However, whether mitoSGs contain preinitiation complexes or indeed any protein is unknown.

While nucleic acid-containing mitochondrial stress granules have not been previously described, mitochondrial RNA granules have been observed in various immortalized cell lines [49]. Mitochondrial RNA granules have been hypothesized to function as organizing centers for mitochondrial RNA processing and translation and are closely associated with but physically and biochemically distinct from the nucleoid [49–54]. Crosslinking, immunofluorescence, and fluorescent-tagging studies have identified a large number of proteins that are associated or colocalized with mitochondrial RNA granules [49]. Core nucleoid proteins are infrequently colocalized with and are generally not part of the mitochondrial RNA granule. Twinkle has been found in proximity labelling studies to label some mitochondrial RNA granule proteins, and knockdown of Twinkle can reduce the number of mitochondrial RNA granules [55, 56]. However, depletion of mitochondrial single-stranded DNA binding protein (mtSSB), another nucleoid protein, was found to increase mitochondrial RNA granules [56]. This is in contrast to the mitoSGs described here that were eliminated by the knockdown of both Twinkle and mtSSB, as well as by the knockdown of three other nucleoid proteins. This distinct dependence of the mitoSGs on multiple nucleoid components versus the previously described mitochondrial RNA granules argues that the two granules are not identical. Certainly, the mitochondrial stress granules are induced by stress, whereas mitochondrial RNA granules appear to be constitutive and part of normal mitochondrial function, at least in immortalized cell lines.

Interestingly, double-stranded RNA has been shown to be produced in the mitochondria of human cell lines and is thought to be rapidly degraded by an ATP-dependent helicase and polynucleotide phosphorylase in the mitochondrial RNA degradosome, which is thought to be located near but potentially distinct from the mitochondrial RNA granule [49, 57–60]. Loss of helicase or polynucleotide phosphorylase function can result in the accumulation of mitochondrial double-stranded RNA [57]. Thus, a reasonable hypothesis is that dysfunction of the degradosome by ATP depletion during hypoxia results in the accumulation of double-stranded RNA in the mitochondria that are visualized as MSG. However, how loss of function of LONP1 and CLPP1 proteases might affect the degradosome is not clear.

The effect of the mitochondrial stress granules on mitochondrial function after hypoxia is unclear. Our data did not determine whether the mitochondrial stress granules are deleterious, protective, or neither after stress. If mitoSG formation inexorably leads to cell death, then blocking their formation would be expected to confer resistance to hypoxic death. However, genetic manipulation of nucleoid components that blocked mitoSGs did not consistently alter hypoxic sensitivity. Only *atad-3* and *twnk-1* RNAis were hypoxia protective; it will be interesting to dissect the mechanism whereby these nucleoid components specifically regulate hypoxic death. On the other hand, two mechanistically distinct hypoxia protective pathways blocked mitoSG. Thus, mitoSG seems likely to be either a dysfunctional consequence of hypoxia that is ameliorated in the hypoxia-resistant mutants, or the formation of mitoSG is an adaptive yet dispensable response that is not produced in the hypoxia-protective mutants.

A clue to the functional consequence of mitoSGs might be in the observation that they form in cells lacking the mitochondrial protease LonP. Loss-of-function mutations in *C. elegans lonp-1* increase both misfolded protein stress, oxidized proteins, and overall oxidate stress [39, 61]. In Drosophila, the knockdown of LonP reduces respiratory chain complex catalytic activity and assembled complex levels [62]. This effect on the respiratory chain may be secondary to an observed marked reduction in the translation of mitochondrial transcripts. In LonP knockdown flies, mitochondrial transcripts were found to sediment as large particles, consistent with aggregation of mitochondrial transcripts causing a reduction in their translation [62]. Interestingly, overexpression of fly ClpP protease ameliorated behavioral defects in the fly produced by LonP knockdown. This result is consistent with our finding that the knockdown of *C. elegans* ClpP also produces ethidium bromide staining granules resembling mitoSGs. In yeast, mammalian cells, and in human patients, reduction of function of LonP1 results in electron-dense granules in the mitochondrial matrix that may represent mitoSGs although there was no evidence for or against the presence of nucleic acid in these granules [63–65]. Our data combined with these previous studies suggest the hypothesis that hypoxia reduces the ATP-dependent activity of LONP1 and/or ClpP thereby resulting in aggregated mitochondrial transcripts. The effect of these aggregates, at least those produced by the absence of LonP1, would be to reduce mitochondrial translation, reduce respiratory chain activity, and presumably would be deleterious to cellular health. Identifying mechanisms that can specifically target mitochondrial transcript aggregation may lead to promising new strategies to ameliorate early pathological consequences of hypoxic injury.

## Materials and Methods

### C. elegans strains and culture methods

*C. elegans* strains were cultured and maintained at 20 °C, on NGM agar with OP50 *E. coli* food unless otherwise noted [66]. The N2 (Bristol) strain was the standard wild-type strain from the *C. elegans* Genetics Center (CGC, University of Minnesota). The mitochondrial UPR reporter strain SJ4100 (*zcIs13*[*hsp-6*p::GFP]) [67], NL3630 (*pkIs32*[*pie-1*p::GFP::H2B]) [68], SJ4143 (*zcIs17* [*ges-1*p::GFP(mt)]) [69], *ccIs4251* [(pSAK2) *myo-3*p::GFP::LacZ::NLS + (pSAK4) *myo-3*p:: mitoGFP + *dpy-20*(+)] [16], were obtained from the CGC; *lonp-1*(*tm5171*) and *clpp-1*(*tm6212*) were gifts from Professor Y Kohara, National Institute of Genetics, Mishima, Japan. *gcIs46* [*ucr-11*::GFP] and *atfs-1*(*tm4919*);*gcIs46* were generated as previously described [8]. *lonp-1*(*tm5171*);*zcIs46* and *clpp-1*(*tm6212*);*gcIs46* were generated in this study. *lonp-1*(*tm5171*) and *clpp-1*(*tm6212*) were outcrossed with wild-type N2 at least 4 times prior to building these strains and testing.

### Hypoxic incubations and hypoxic injury scoring

Synchronized young adult worms were subjected to hypoxia as described previously [15, 70]. Briefly, each plate of worms was washed into one 1.5 ml tube with 1 ml of M9 buffer (22 mM KH_2_PO_4_, 22 mM Na_2_HPO_4_, 85 mM NaCl, 1 mM MgSO_4_). Worms were allowed to settle by gravity, and 900 μl of M9 was removed. The tubes were then placed in the anaerobic chamber (Forma Scientific) at 26.5 °C for incubation times ranging from 12 to 24 hours as indicated. Oxygen tension was always ≤ 0.3%. Following the hypoxic insult unless otherwise noted, worms were transferred using glass Pasteur pipettes onto NGM plates spotted with OP50 bacteria and recovered at 20 °C for 24 hours. Normoxic incubations were otherwise identical except performed in a 26.5 °C room air incubator. Organismal death was scored as described [70]. Briefly, animals were scored as dead if pharyngeal pumping, spontaneous and evoked movement (touching with a platinum wire) were not observed. For aggregates assay, synchronized worms were kept on NGM plates and placed into anaerobic chamber for 2 to 15 hours as stated in the figure legend.

### RNAi experiments

Control RNAi strain, L4440 (empty vector), *hmg-5*(RNAi), *atad-3*(RNAi), *mtss-1*(RNAi), *polg-1*(RNAi) and *twnk-1*(RNAi) were from the Ahringer *C. elegans* RNAi library (MRC Gene service) [71]. *atfs-1*(RNAi) strain was from Vidal ORF-RNAi *C. elegans* RNAi library [72] (a kind gift from Dr. T. Schedl, Washington University in St. Louis, MO). Bacterial clones containing RNAi plasmids were cultured and induced with 0.1% β-lactose in 100 mg/ml ampicillin for 24 hr at 23 °C [73]. Worms were synchronized on RNAi plates for 3.5 days (N2) or 4.5 days (animals in a *lonp-1(tm5171)* background) until reaching adulthood; worms not reaching adulthood were excluded.

### Mitochondrial UPR reporter and screening

SJ4100 (*zcIs13*[*hsp-6p*::GFP]) was used as a reporter for mitochondrial UPR [30]. Animals were synchronized from eggs by bleaching gravid adults and incubated on indicated RNAi plates for 3 days until adulthood. The GFP expression were imaged, and intensity of fluorescence was determined by ImageJ (NIH).

#### Chemical treatment and EtBr and SYBR gold staining

Doxycycline, dissolved in DMSO (Dimethyl sulfoxide), was diluted with 250 μL nuclease-free, deionized water and spread evenly to a final concentration of 100 mM. Worms were synchronized on the plates by bleaching and grew for 3.5 days to 1-day-old adults. EtBr (ThermoFisher Scientific)- and SYBR gold (Invitrogen)-containing plates were made the same way as described above with a concentration of 8 µg/ml ( ~ 20 µM) and a 1/5000 dilution of SYBR gold 10,000X concentrate as supplied by Invitrogen, respectively. For staining, 1-day-old synchronized worms were transferred onto ethidium bromide or SYBR-gold-containing plates for 8 to 24 hours. Worms were then transferred on the plates into the hypoxic chamber with a chamber temperature of 26.5 °C for 12 hours or transferred to a normoxic incubator at 26.5 °C. After removing worms from the hypoxic or normoxic incubators, animals were transferred onto standard agar pads on glass slides for imaging.

#### DNase I and RNase A treatment of nematode gonads and mammalian cells

For *C. elegans*, animals, pre-stained with EtBr (8 µg/ml or 20.3 µM) and SYBR gold (1/5000) followed by hypoxia, were gonad dissected, fixed (4% formaldehyde, Electron Microscopy Sciences), permeabilized (PBS + 0.1% triton-X 100, Sigma), and treated with DNase I (50 U/ml, New England BioLabs) or RNase A (500 mg/ml, New England BioLabs). The gonads were then mounted and imaged.

For mammalian cells, the H9c2, embryonal rat heart-derived cells were obtained from the ATCC (atcc.org) and were cultured in Iscove’s Modified Dulbecco’s Medium (IMDM, Gibco) supplemented with 10% fetal calf serum (Gibco). The cells grown on glass cover slips, with less than 80% confluence, were prestained with EtBr (1.58 µg/ml or 4 µM) and SYBR gold (1/10,000X) or MitoTracker (0.25 µM) (Cell Signaling) followed by sodium azide (NaN_3_, 100 mM, 3 h, Sigma) treatment. For Mitotracker/EtBr staining, cells were immediately mounted and imaged by confocal microscopy. For DNase and RNAse experiments, cells were fixed (4% formaldehyde, Electron Microscopy Sciences), permeabilized (PBS + 0.1% triton-X 100, Sigma), and treated with DNase I (50 U/ml, New England BioLabs) or RNase A (500 mg/ml, New England BioLabs). The cells were then mounted and imaged.

### Light microscopy

Images of UCR-11::GFP aggregates and TMRE signals were acquired with confocal microscopy as previously described [8, 11]. Briefly, paralysis was produced by mounting worms in a solution of 50 mM levamisole (Sigma-Aldrich Corp., St. Louis, MO, USA) in M9 prior to imaging. Images were acquired from at least 5–10 randomly selected worms at 1024 × 1024 resolution using a 63 × objective with 8 × zoom producing a 23.07 × 23.07 μm image (defined as one high power field, HPF). All images were acquired as a ten-slice Z-stack with scan speed of 800–1800 Hz and flattened as a maximum intensity projection prior to analysis. The wavelength of the laser to capture the signal for SYBR gold/GFP and EtBr signals was 500–530 nm and 620–700 nm, respectively. For assessment of total fluorescence images were acquired on a Zeiss Axioskop2 microscope with fluorescence intensity quantified using NIH ImageJ.

#### Determination of relative mt/nuclear DNA content

Power SYBR Green PCR Master Mix (Applied Biosystems) was used for the quantitative PCR with 7500 Fast Real Time PCR System (Applied Biosystems) to determine mitochondrial DNA content relative to nuclear DNA content as previously described [74]. Briefly, for the amplification of *C. elegans* mitochondrial DNA (75 bp), the following primer sets were used: nd-1 forward, 5’- AGCGTCATTTATTGGGAAGAAGAC -3’, nd-1 reverse, 5’- AAGCTTGTGCTAATCCCATAAATGT -3’. For the amplification of *C. elegans* nuclear DNA (164 bp), the following primer sets were used: Cox-4 forward, 5’- GCCGACTGGAAGAACTTGTC-3’, Cox-4 reverse, 5’-GCGGAGATCACCTTCCAGTA-3’. The *C. elegans* DNA template used for the quantitative PCR was prepared as follows. The worms were incubated with lysis buffer (20 mM Tris pH 7.5, 50 mM EDTA, 200 mM NaCl, 0.5% SDS and Proteinase K to 100 μg/ml) for 1 h at 60 °C, and then the proteinase reaction was inactivated by incubation for 10 min at 95 °C. Next, the lysate was appropriately used as the template. Relative mitochondrial DNA copy number was normalized with nuclear DNA. For RNAi experiments, worms were synchronized and grown on indicated RNAi plates until day 0 adults. *hmg-5*(*tm3586*) were picked from synchronized *hmg-5*(*tm3586)/nT1* grown on OP50/NGM plates. For hypoxia experiments, synchronized young adult worms were incubated in hypoxia for 14 h on plates and recovered for 1 h at room temperature then surviving worms were lysed for qPCR as described above.

### Statistics

Two-sided unpaired t-tests with the assumption of unequal variance was used for statistical comparisons; the normality of data was not formally assessed. Statistics were calculated using GraphPad Prism 6.01 (San Diego, CA, USA) and Excel 2007 (Microsoft, Redmond, WA). Values are expressed as mean ± SD. A P value of ≤ 0.05 was considered significant. For comparative statistics (Figs. 4–7, Suppl. Fig. 5), samples sizes were chosen based on preliminary estimates of the environmental variance of the data and ranged from 3-20 independent replicates. Sample sizes with the number of independent replicates and technical replicates are stated in figure legends with independent replicates shown as individual data points shown for all quantitative data except hypoxic death experiments (Fig. 4f). Statistics were performed after data was collected in full and the number of replicates performed was not based on statistical significance calculations. The investigators were not blinded to group allocation given that endpoints scored were not subject to observer bias. Randomization was not performed given that individuals within groups are genetically and environmentally identical. All replicates/animals were included in analysis; none were excluded.

## Supplementary information

Suppl Fig 1

Suppl Fig 2

Suppl Fig 3

Suppl Fig 4

Suppl Fig 5

Suppl Fig 6

Suppl Fig 7

Suppl Fig 8

Suppl Fig Legends

aj-checklist

## Data Availability

The raw datasets generated and analyzed during the current study are available from the corresponding author on reasonable request.
