# Supplementary figures and images for: Hypoxia-induced mitochondrial stress granules
Source: Cell Death Dis. 2023 Jul 19;14(7):448. doi: 10.1038/s41419-023-05988-6 (PMC10356818; doi:10.1038/s41419-023-05988-6)

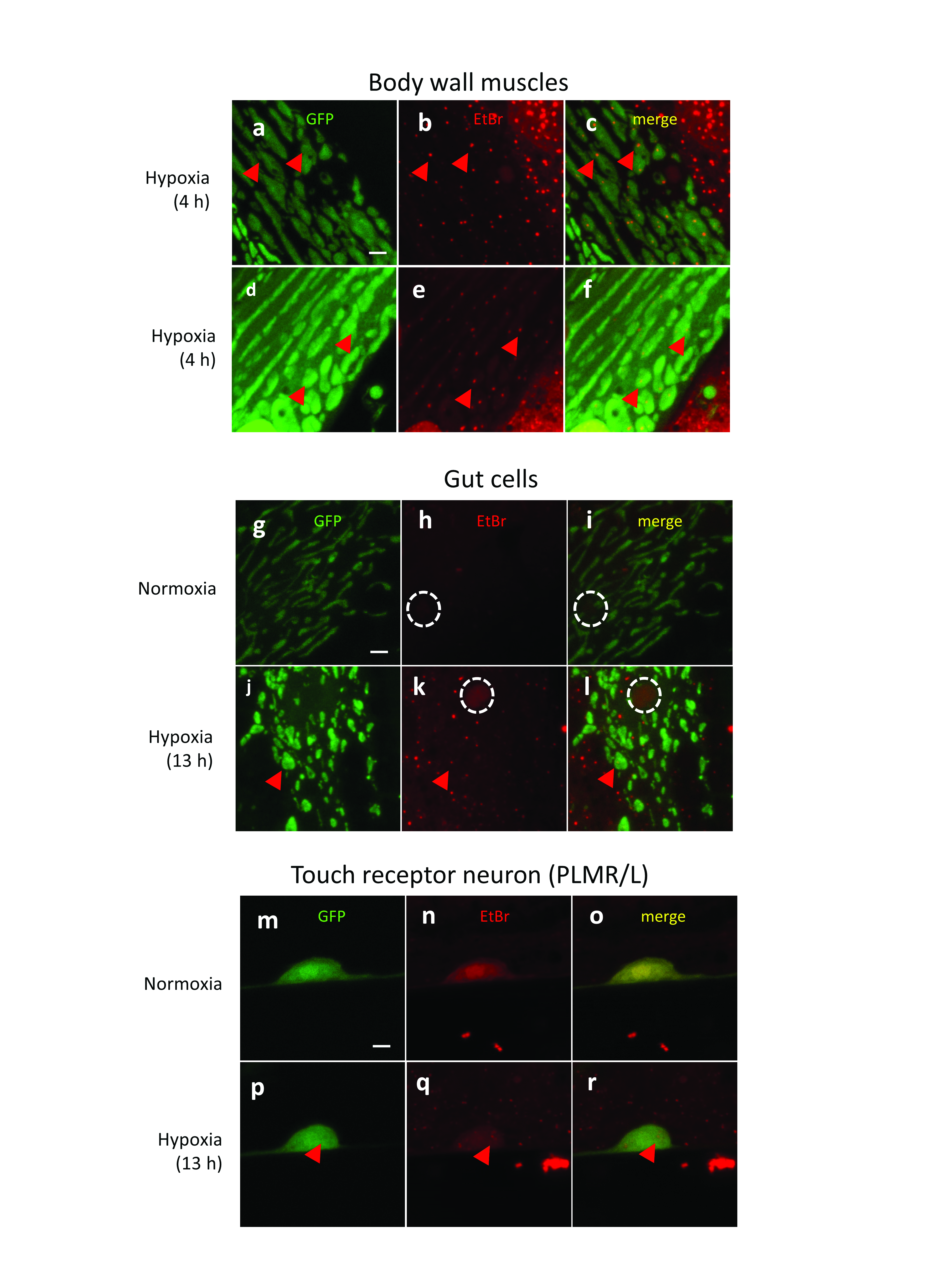

Supplement: Supplementary file 2 — Suppl Fig 2 [file 41419_2023_5988_MOESM2_ESM.tif]

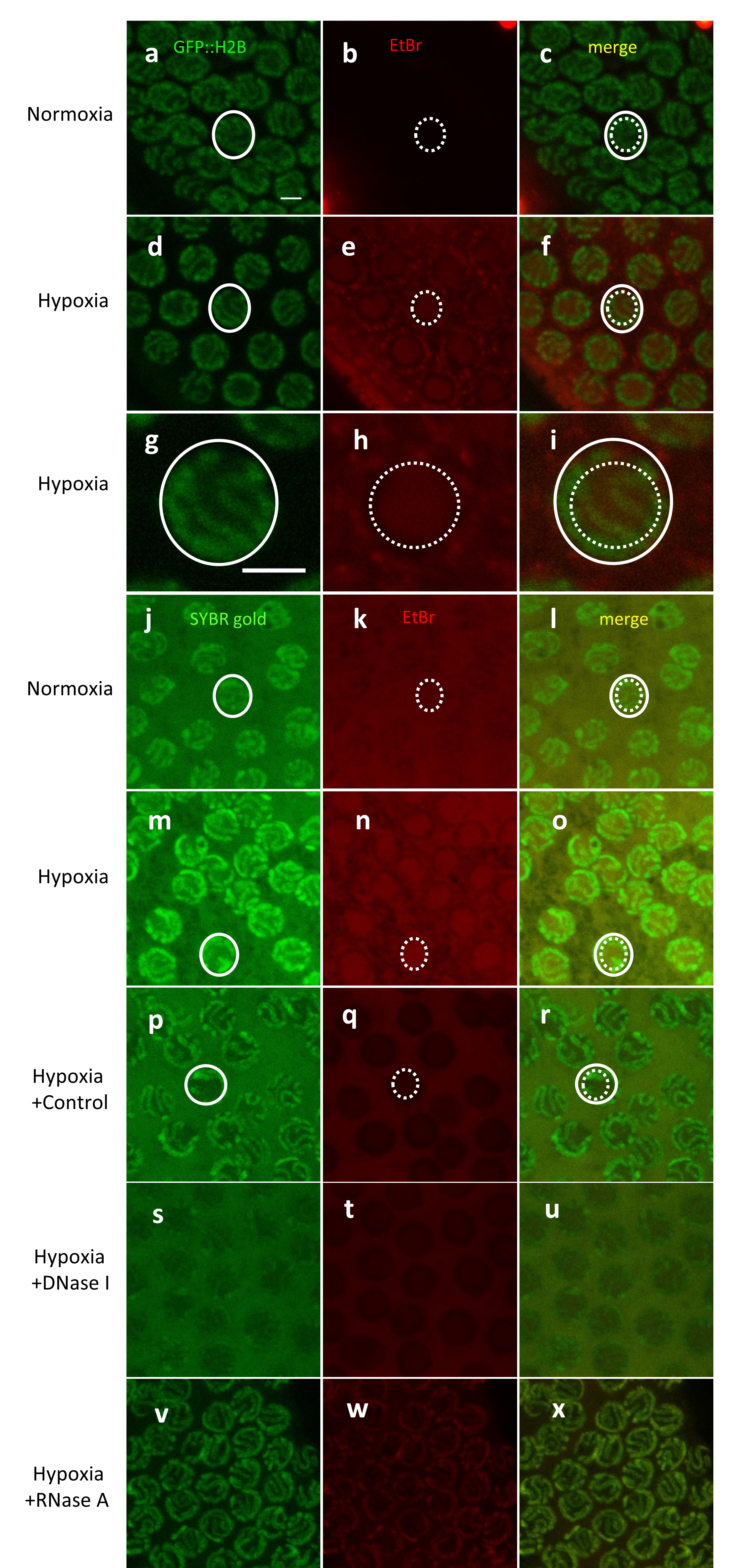

Supplement: Supplementary file 3 — Suppl Fig 3 [file 41419_2023_5988_MOESM3_ESM.tif]

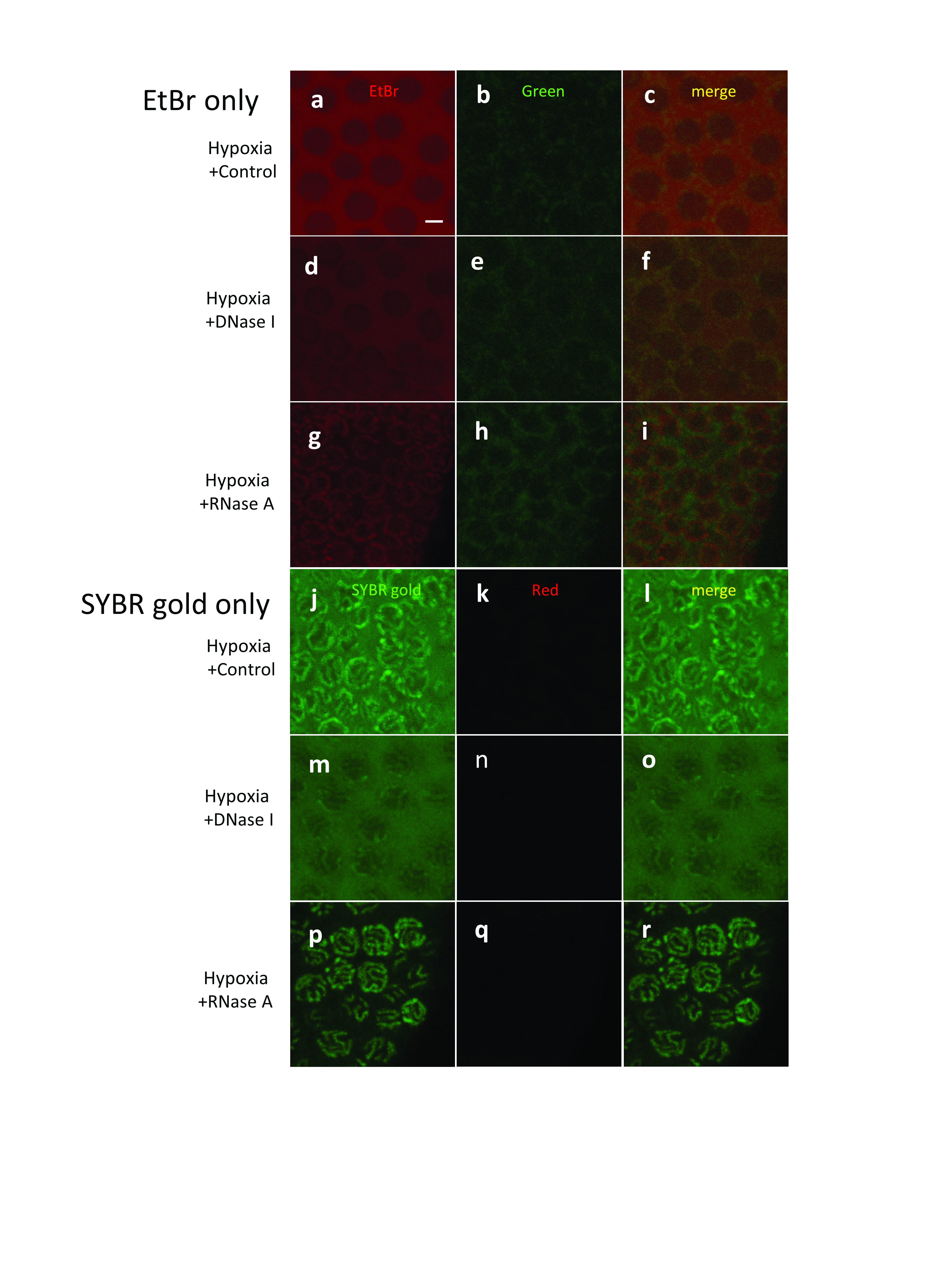

Supplement: Supplementary file 4 — Suppl Fig 4 [file 41419_2023_5988_MOESM4_ESM.tif]

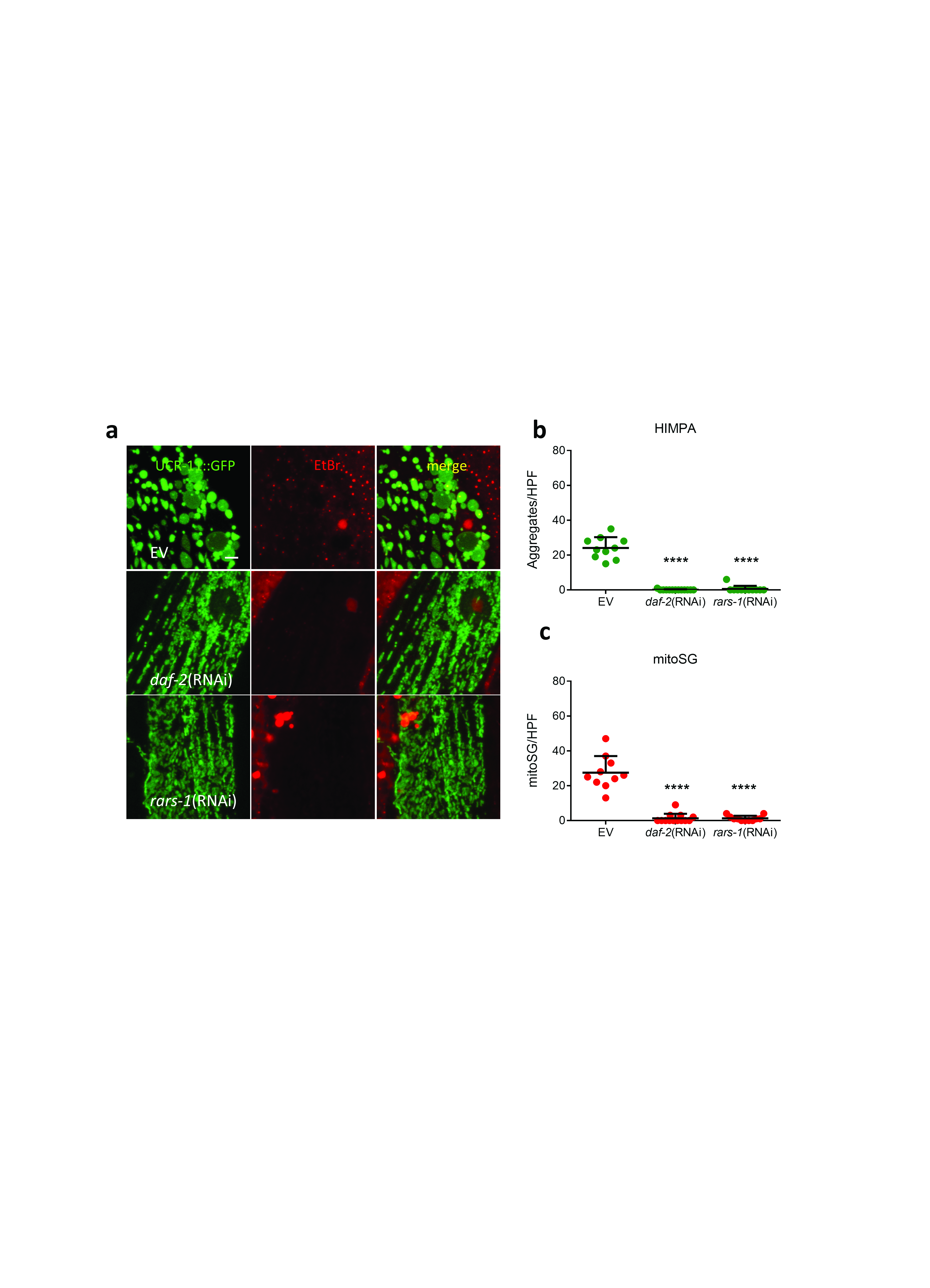

Supplement: Supplementary file 5 — Suppl Fig 5 [file 41419_2023_5988_MOESM5_ESM.tif]

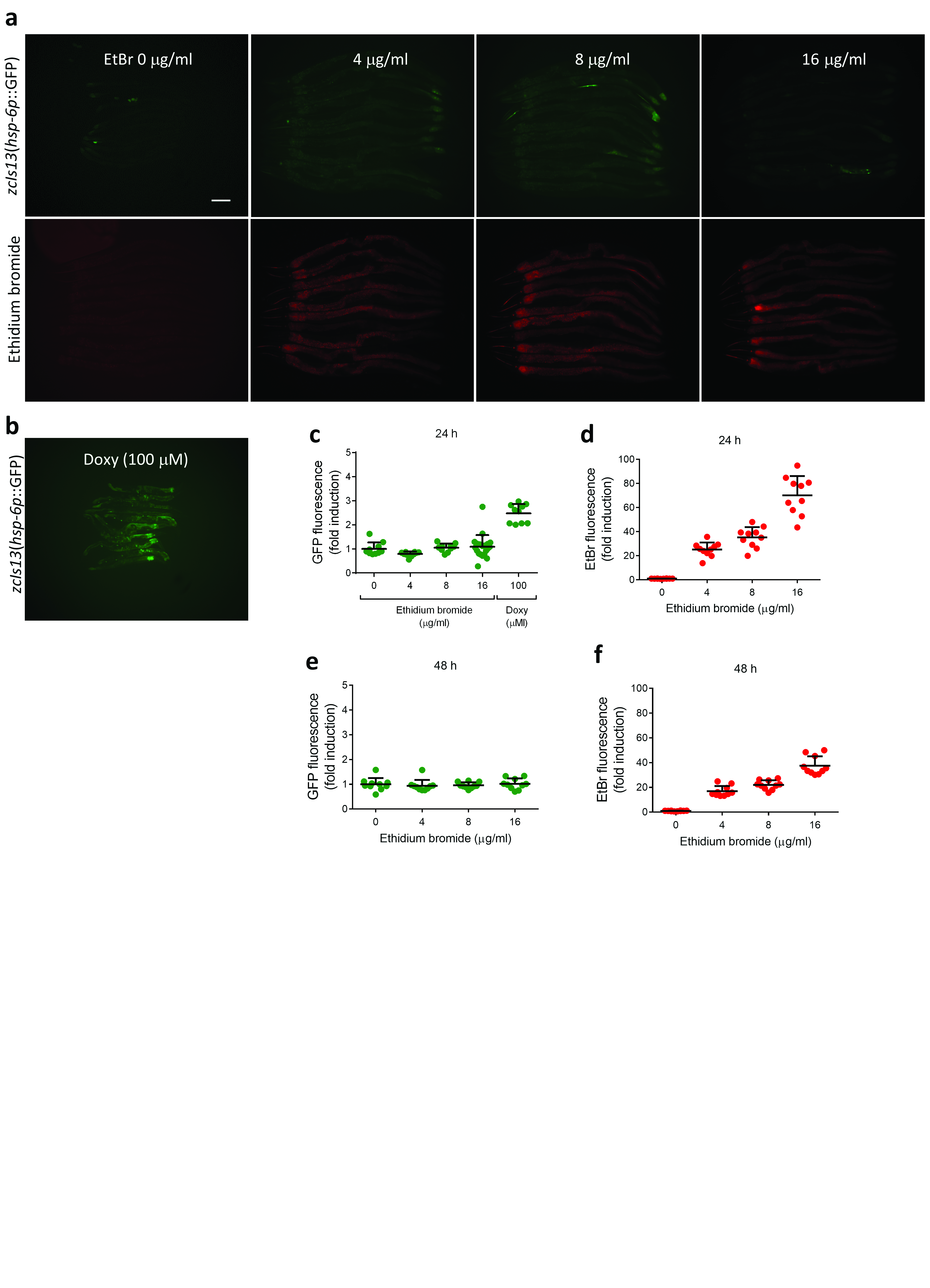

Supplement: Supplementary file 6 — Suppl Fig 6 [file 41419_2023_5988_MOESM6_ESM.tif]

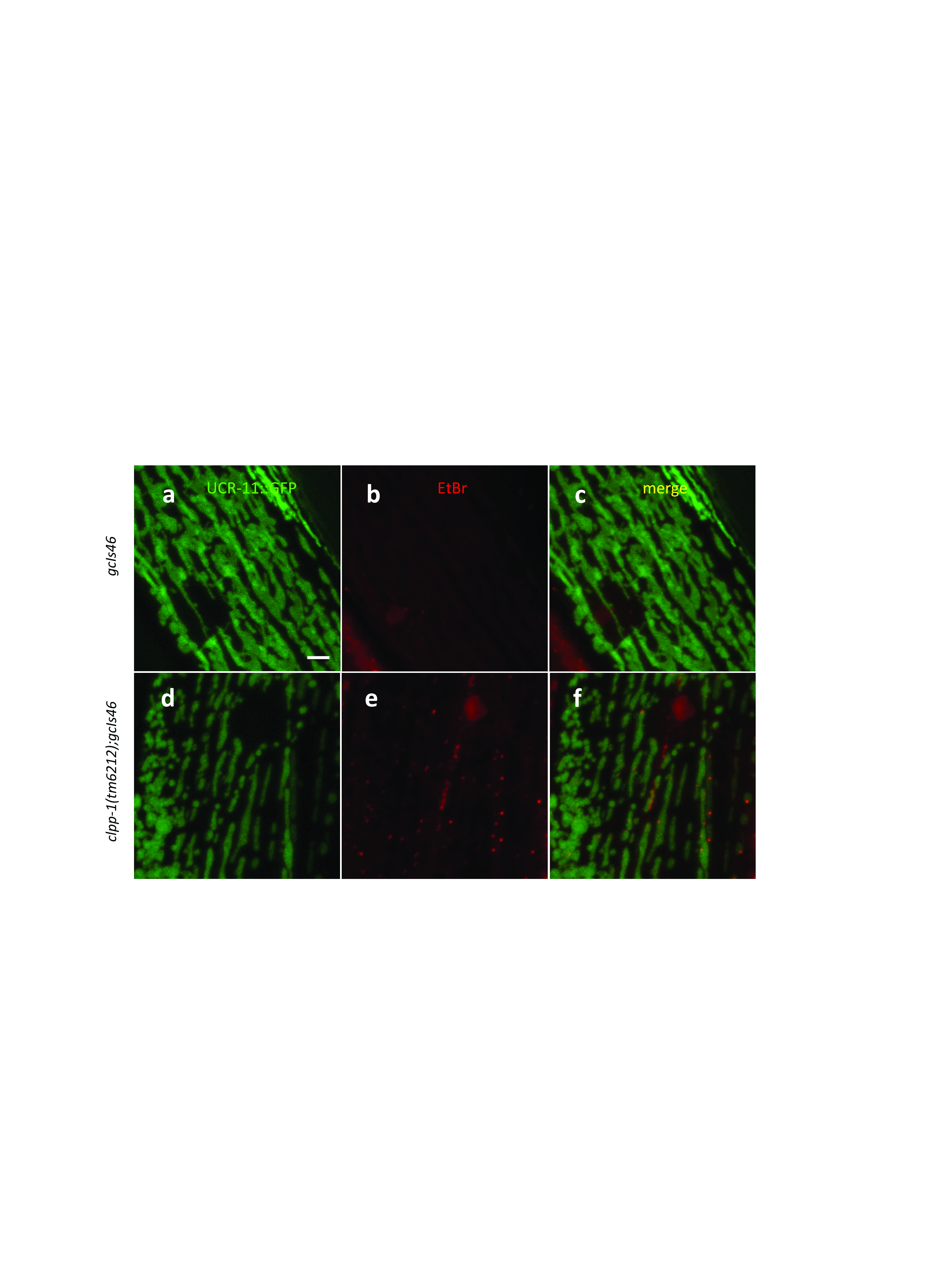

Supplement: Supplementary file 7 — Suppl Fig 7 [file 41419_2023_5988_MOESM7_ESM.tif]

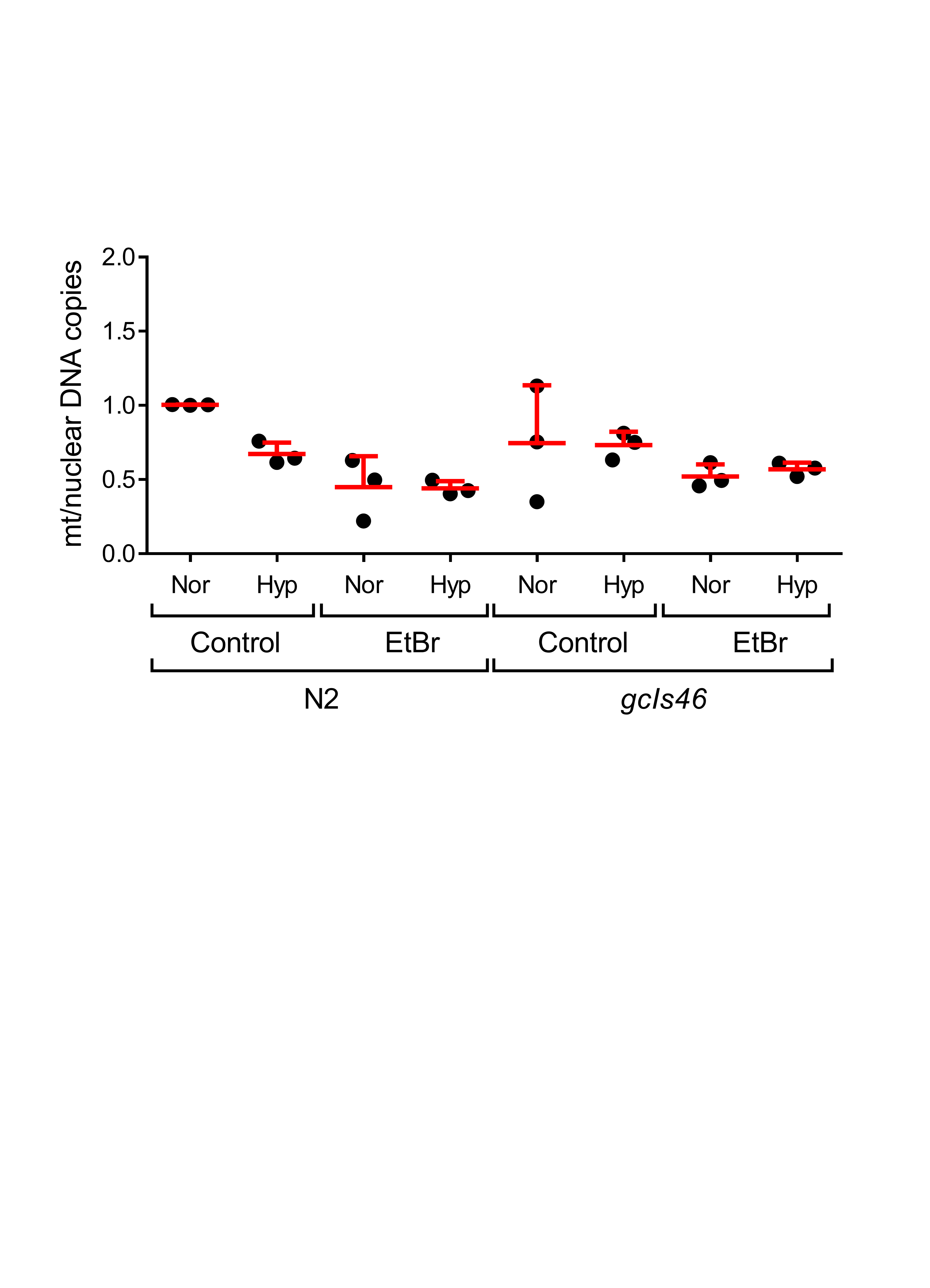

Supplement: Supplementary file 8 — Suppl Fig 8 [file 41419_2023_5988_MOESM8_ESM.tif]
