# Supplementary material for: Hypoxia-induced mitochondrial stress granules
Source: Cell Death Dis. 2023 Jul 19;14(7):448. doi: 10.1038/s41419-023-05988-6 (PMC10356818; doi:10.1038/s41419-023-05988-6)
Supplement: Supplementary file 9 — Suppl Fig Legends [file 41419_2023_5988_MOESM9_ESM.docx]

**Supplementary figure 1.** **Movie of EtBr-staining stress granules and HIMPA.** 10 image Z-stack movie of UCR-11::GFP HIMPA (green) and EtBr staining mitoSGs (red) imaged immediately after 12h hypoxia incubation.

**Supplementary figure 2. EtBr-staining stress granules not specific to UCR-11 expressing cells or to muscle cells. (a-f)** Hypoxia-induced EtBr staining mitoSGs in body wall muscles. **(a-c)** and in *ccIs4251* (*myo-3*p:: mitoGFP) animals **(d-f)**. **(g-l)** Hypoxia-induced EtBr staining mitoSGs in intestinal cells. EtBr staining after normoxia **(g-i)** or hypoxia (13 h) **(j-l)** in *zcIs17* [*ges-1*p::GFP(mt)] animals. EtBr staining in nucleolus (dotted white circle). **(m-r)** Hypoxia-induced EtBr stains in touch receptor neurons. EtBr stains after normoxia **(m-o)** or hypoxia (13 h) **(p-r)** in *zdIs5 [mec-4p::GFP + lin-15(+)]* animals. Scale bar = 3 μm. Red arrowhead indicates location of an EtBr staining granule.

**Supplementary Figure 3. EtBr stains RNA in germ cells. (a-f)** Nuclear localized GFP::H2B (*pkIs32*[*pie-1p:*:GFP::H2B]) and EtBr staining after 14h normoxia **(a-c)** or 14 h hypoxia **(d-f). (g-I)** 3-fold magnification of **d-f.** GFP::H2B-labeled nuclear DNA (white circle) and EtBr stained nucleolus (dotted white circle).  **(j-x)** N2 animals pre-stained with SYBR gold and EtBr for 24 hours and imaged immediately following normoxia **(j-l)** or 14h hypoxia **(m-o)**. SYBR gold-stained nuclear DNA (white circle) and EtBr-stained nucleolus (dotted white circle). **(p-x)**  N2 animals were pre-stained with SYBR gold and EtBr, exposed to 14h hypoxia then immediately fixed, permeabilized, treated with buffer control, DNase I, or RNase A, and imaged. Treatment without **(**Control, **p-r)** or with DNase I **(s-u)** or RNase A **(v-x)**. Scale bar = 3 μm.

**Supplementary figure 4. Single dye staining shows crossover staining is not bleed-through. (a-i)** N2 animals were pre-stained with EtBr only followed by 14 h hypoxia. Non-treatment **(a-c)** or treatment with DNase I **(d-f)** or RNase A **(g-i)**. Red channel (EtBr), green channel (Green), and merged (merge) images shown. **(j-r)** N2 animals were pre-stained with SYBR gold only followed by 14 h hypoxia. Non-treatment **(j-l)** or treatment with DNase I **(m-o)** or RNase A **(p-r)**. Green channel (SYBR gold), red channel (Red) and merged (merge) images. Scale bar = 3 μm.

**Supplementary Figure 5. Hypoxia resistance blocks mitoSG formation. (a)** Examples of UCR-11::GFP HIMPA (green) and EtBr staining mitoSGs (red) in animals grown on empty vector (EV), *daf-2*(RNAi) or *rars-1*(RNAi) scored immediately after 12 h hypoxia. Scale bar = 3 μm. **(b).** Quantification of UCR-11::GFP HIMPA. **(c)** Quantification of mitoSGs. Mean ± SD from 10 animals from 2 independent experiments. **** p<0.0001 by unpaired, 2-sided t-test versus EV.

**Supplementary figure 6. EtBr treatment does not induce UPR^mt^** Expression of hsp-6p::GFP is a reporter of activation of the UPR^mt^. **(a)** *zcIs13*(*hsp-6*p::GFP) animals incubated without or with indicated EtBr concentrations for 24 hours. GFP expression and EtBr staining imaged. **(b)** *zcIs13*(*hsp-6*p::GFP) animals incubated with doxycycline for 24 hours. **(c,e)** Quantification of GFP expression or **(d,f)** EtBr staining for 24 **(c,d)** or 48 hours **(e,f)**. Data are mean ± SD from 10 animals. Scale bar=0.1mm.

**Supplementary figure 7. Loss-of-function of *clpp-1* induces mitoSGs. (a-f)** UCR-11::GFP HIMPA and EtBr staining mitoSGs in normoxia in *gcIs46* in a wild type background **(a-c)** and in a *clpp-1*(*tm6212*) background **(d-f)**. Scale bar = 3 μm.

**Supplementary figure 8. Hypoxia does not increase mtDNA copy number.** Mitochondrial DNA copy number relative to nuclear DNA in young animals exposed to 0 (Control) or 8 mg/ml ethidium bromide (EtBr) for 24 hours. Animals were then exposed to 14 hours of hypoxic or normoxic incubation, recovered for one hour then lysed for quantitative PCR analysis. Each data point represents an independent biological replicate with 4 technical replicates averaged per point. Data summarized as mean ± SD.
